# Transforming personality traits (*Gunas*) through meditation: A randomized controlled trial

**DOI:** 10.6026/973206300211340

**Published:** 2025-06-30

**Authors:** Chanchal Surywanshi, Kawal Krishan, Danish Javed

**Affiliations:** 1 Department of Integrative Medicine, the Sri Devaraj Urs Academy of Higher Education and Research Kolar, Karnataka, India; 2 Department of Hospital Administration, All India Institute of Medical Sciences, Bhopal, Madhya Pradesh, India; 3 Department of AYUSH, All India Institute of Medical Sciences, Saket Nagar, Bhopal, Madhya Pradesh, India

**Keywords:** Yoga, meditation, personality traits, randomized controlled trial, stress, psychological, medical students

## Abstract

Medical students often experience stress and maladaptive personality traits that affect mental health. This randomized controlled trial evaluated the impact of a six-week Trimurti *Dhyana* (Meditation) Yoga intervention on personality traits (*Gunas*) in 70 male medical students aged 18-30 years. The intervention group practiced daily meditation for 20 minutes, while the control group continued routine activities. Post-intervention, the meditation group showed a significant increase in *Sattva* and reductions in *Rajas* and *Tamas* scores as measured by the Vedic Personality Inventory. These findings suggest that *Trimurti Dhyana Yoga* is an effective, non-invasive strategy for enhancing psychological resilience in high-stress populations.

## Background:

Yoga, with its origins in ancient Indian philosophy, is increasingly recognized as a scientifically validated approach to improving physical, mental and emotional well-being [1]. Rooted in holistic principles, yoga integrates physical postures, breath control and meditation to promote a balanced state of mind and body [2]. Among the many dimensions of yoga, Dhyana (meditation) holds a pivotal place in fostering self-awareness, enhancing cognitive functioning and mitigating stress [3]. *Trimurti Dhyana*, a meditative practice based on the triadic concept of the *Hindu* deities-*Brahma* (creator), *Vishnu* (preserver) and *Shiva* (transformer)-offers a unique perspective by aligning cognitive and emotional states with the principles of creation, sustenance and transformation [4]. Personality, as described in classical Indian texts like the *Vedas* and *Upanishads*, is shaped by the interplay of three fundamental attributes, or *Gunas*: *Sattva* (associated with purity, harmony and wisdom), *Rajas* (linked to activity, restlessness and ambition) and *Tamas* (connected to inertia, ignorance and lethargy) [5]. The Vedic framework provides a unique paradigm for personality assessment, emphasizing a dynamic and modifiable interplay of these traits [6]. The Vedic Personality Inventory (VPI), developed by Wolf (1999), has been validated as a reliable tool to quantify these *Gunas* [7]. Previous research has demonstrated the utility of the VPI in both clinical and non-clinical settings, highlighting its sensitivity to interventions that modify cognitive and emotional states [8]. Medical education is inherently rigorous and demanding, often leading to heightened levels of stress, anxiety and burnout among students [9]. The prevalence of psychological distress in medical students is well-documented, with numerous studies reporting adverse effects on their mental health, academic performance and interpersonal relationships [10]. Stress not only impacts their immediate well-being but may also predispose them to long-term psychological and physical health challenges [11].

Evidence from recent studies underscores the importance of mind-body interventions like yoga in mitigating stress and fostering resilience [12]. For instance, systematic reviews and meta-analyses have consistently shown that yoga-based interventions significantly reduce symptoms of anxiety and depression while improving overall quality of life [13]. Specifically, meditation practices have been shown to modulate neurophysiological pathways associated with stress and emotional regulation [14]. Functional MRI studies reveal increased activation in the prefrontal cortex and reduced amygdala activity in individuals practicing meditation, suggesting enhanced emotional control and reduced reactivity to stressors [15]. Additionally, meditation has been associated with favourable changes in autonomic regulation, including reductions in heart rate and cortisol levels, further reinforcing its potential as a stress-relief modality [16]. Therefore, it is of interest to report the impact of a six-week *Trimurti Dhyana Yoga* intervention on personality traits, as measured by the VPI, in a cohort of medical students.

## Material and Methods:

## Methodology:

## Study design:

This study was designed as a prospective, randomized controlled trial to evaluate the effects of a six-week *Trimurti Dhyana Yoga* intervention on personality traits (*Gunas*) among male medical students aged 18-30 years. Participants were randomly assigned to either the Dhyana Group (DG), which underwent the intervention, or the Control Group (CG), which maintained their regular daily activities without exposure to yoga. The study adhered to the Consolidated Standards of Reporting Trials (CONSORT) guidelines to ensure transparency and rigor in reporting.

## Sample size and participants:

A purposive sample of 70 participants was selected for this study and allocated into two groups in a 1:1 ratio. Participants were recruited from Sri Devaraj Urs Academy of Higher Education and Research, Karnataka, India. A total of 70 male students who met the specified inclusion and exclusion criteria were enrolled in the study.

## Inclusion criteria:

[1] Healthy male individuals aged 18-30 years.

[2] Individuals not engaged in regular yoga practice.

## Exclusion criteria:

[1] Regular practitioners of yoga, gym, or sports.

[2] Individuals with spine-related disorders or chronic illnesses.

[3] Participants diagnosed with psychiatric conditions.

[4] Individuals on medications affecting mood or personality traits.

## Ethical considerations:

Ethical approval for the study was obtained from the Institutional Ethics Committee (IEC) (SDUAHER/KLR/CEC/69/2021-22). Written informed consent was secured from all participants prior to enrollment.

## Randomization and blinding:

Randomization was conducted using computer-generated random numbers from www.randomizer.org, allocating 35 participants to the DG and 35 to the CG. Due to the nature of the intervention, blinding of participants was not feasible; however, the researchers and statisticians analyzing the data were blinded to group allocations to minimize bias.

## Intervention:

Participants in the DG underwent a six-week *Trimurti Dhyana Yoga* program. The intervention consisted of 20-minute daily sessions led by certified yoga instructors. The protocol included three phases aligned with the Trimurti concept:

[1] Creation (*Brahma*): Guided visualization to foster creativity and positive intentions.

[2] Preservation (*Vishnu*): Focused breathing exercises to promote balance and stability.

[3] Transformation (*Shiva*): Reflective meditation to encourage adaptive thought processes and emotional resilience.

The CG did not receive any form of yoga intervention and continued with their regular daily activities.

## Outcome measures:

The primary outcome measure was the change in *Gunas* (*Sattva*, *Rajas* and *Tamas*) assessed using the Vedic Personality Inventory (VPI). The VPI is a validated tool with high internal consistency (Cronbach's alpha = 0.84-0.91) and has been widely used in studies assessing personality traits. VPI scores were recorded at baseline (Day 1) and post-intervention (Week 6). Secondary outcomes included self-reported stress levels, assessed using a standardized scale and physiological measures such as resting heart rate and blood pressure, collected at baseline and post-intervention.

## Data collection procedure:

Baseline data were collected on the first day through a structured questionnaire, followed by VPI assessments and physiological measurements. Post-intervention data collection mirrored the baseline protocol, ensuring consistency. The instructors maintained attendance records and monitored adherence to the intervention.

## Sample size calculation:

Sample size was determined based on a pilot study indicating an expected mean difference of 20% in *Sattva* scores between groups, with a standard deviation of 5. Using a significance level of 0.05 and power of 0.80, a sample size of 35 participants per group was calculated to detect a statistically significant difference.

## Statistical analysis:

Data were analyzed using SPSS software (Version 25.0). Descriptive statistics summarized demographic and baseline characteristics. Paired t-tests assessed within-group changes and independent t-tests compared between-group differences. Effect sizes were calculated using Cohen's d. A p-value of <0.05 was considered statistically significant.

## Results:

A total of 70 male participants were enrolled and randomly allocated into the Dhyana Group (DG; n = 35) and the Control Group (CG; n = 35) (Figure 1 - see PDF). The mean age of participants was 21.71 ± 3.48 years in the DG and 21.80 ± 2.75 years in the CG, with no statistically significant difference between groups (p = 0.91). Baseline comparisons of *Sattva*, *Rajas* and *Tamas* scores using independent t-tests showed no significant differences (p > 0.05), confirming the homogeneity of groups at the start of the study. Paired t-tests were conducted to assess within-group changes in *Sattva*, *Rajas* and *Tamas* scores. Between-group comparisons were analyzed using ANCOVA, adjusting for baseline scores to account for any residual confounding. Effect sizes (Cohen's d) were computed to evaluate the magnitude of the intervention's impact. In the DG, the *Sattva* score significantly increased from 37.25 ± 4.84 to 47.45 ± 5.98 (mean difference = 10.20, p < 0.001), indicating a 27.38% improvement. No significant change was observed in the CG (38.04 ± 5.59 to 38.03 ± 4.64; p = 0.971). ANCOVA revealed a statistically significant difference between groups post-intervention (F (1, 67) = 45.32, p < 0.001), with the DG showing a higher adjusted mean score. The effect size was large (Cohen's d = 1.87) (Table 1 - see PDF, Figure 2 - see PDF). The *Rajas* score in the DG decreased significantly from 32.68 ± 2.31 to 29.24 ± 3.97 (mean difference = -3.44, p < 0.001), reflecting a 10.52% reduction. The CG showed no significant change (32.73 ± 3.69 to 32.81 ± 3.16; p = 0.728). Adjusted post-intervention scores were significantly lower in the DG than in the CG (F (1, 67) = 39.87, p < 0.001). The effect size for the reduction in *Rajas* was moderate to large (Cohen's d = 1.05) (Table 2 - see PDF, Figure 3 - see PDF).

The DG exhibited a significant reduction in *Tamas* scores from 30.06 ± 4.28 to 23.31 ± 3.84 (mean difference = -6.75, p < 0.001), equating to a 22.45% improvement. The CG showed no significant change (29.24 ± 3.96 to 29.16 ± 3.09; p = 0.797). Post-intervention adjusted scores were significantly lower in the DG compared to the CG (F (1, 67) = 52.13, p < 0.001), with a large effect size (Cohen's d = 1.66) (Table 3 - see PDF, Figure 4 - see PDF). Secondary analyses revealed significant reductions in stress levels (p < 0.001) and resting heart rate (p = 0.002) in the DG, with no corresponding changes in the CG. Blood pressure changes were minimal and not statistically significant across groups. The six-week *Trimurti Dhyana Yoga* intervention led to a statistically significant improvement in *Sattva* scores and reductions in *Rajas* and *Tamas* scores compared to the control group. These changes suggest a shift toward adaptive personality traits and reduced stress. Effect sizes across all domains indicated moderate to large impacts, reinforcing the robustness of the intervention. To further explore the robustness of findings, a sensitivity analysis using non-parametric tests (Mann-Whitney U for between-group and Wilcoxon signed-rank for within-group comparisons) confirmed the significance of results (p < 0.001 for all comparisons). This randomized controlled trial aimed to evaluate the effects of a six-week *Trimurti Dhyana Yoga* intervention on personality traits (*Gunas*) as measured by the Vedic Personality Inventory (VPI) in medical students. The results demonstrated significant improvements in *Sattva* scores and reductions in *Rajas* and *Tamas* scores in the intervention group compared to the control group, suggesting that *Trimurti Dhyana Yoga* positively influences personality traits.

## Discussion:

The findings of this study align with prior research emphasizing the psychophysiological benefits of yoga and meditation. Meditation practices have been shown to modulate brain regions associated with self-regulation and emotional processing, including increased activation in the prefrontal cortex and decreased amygdala activity [17]. Such changes likely underpin the observed increase in *Sattva* and the reductions in *Rajas* and *Tamas* scores, reflecting enhanced emotional stability and reduced reactivity to stress. Studies investigating the impact of yoga on stress and personality traits have consistently reported similar trends. For instance, Goyal *et al.* (2014) conducted a systematic review and meta-analysis, highlighting significant reductions in anxiety and depression among individuals practicing meditation [18]. This evidence supports the current study's findings of reduced *Rajas* and *Tamas*, traits often associated with heightened emotional distress and inertia, respectively.

The observed changes in personality traits can be partially attributed to the physiological effects of meditation on the hypothalamic-pituitary-adrenal (HPA) axis and autonomic nervous system. Meditation practices, including *Trimurti Dhyana*, have been linked to reductions in cortisol levels and sympathetic nervous system activity [19]. These changes are known to enhance emotional regulation and decrease impulsive or maladaptive behaviours, potentially explaining the reductions in *Rajas* and *Tamas*. Additionally, the focus on the Trimurti concept-creation, preservation and transformation-may have a unique cognitive and emotional impact by encouraging participants to adopt a balanced perspective on life's challenges. This integrative approach could foster an adaptive shift toward *Sattva*, characterized by clarity, harmony and mindfulness. Medical students are particularly vulnerable to stress and burnout due to their demanding academic and clinical responsibilities [20]. High stress levels have been linked to maladaptive personality traits and poor mental health outcomes [21]. Interventions such as *Trimurti Dhyana Yoga* provide a holistic method to address these challenges, promoting not only stress reduction but also the cultivation of traits conducive to resilience and professional success. The significant increase in *Sattva* and reduction in *Rajas* and *Tamas* suggest that this intervention can help medical students achieve greater emotional balance, cognitive clarity and overall well-being. Such changes may translate into improved academic performance, better interpersonal relationships and enhanced patient care in their future careers [22]. A major strength of this study is its rigorous design, including randomization, the use of validated assessment tools and the inclusion of both statistical and effect size analyses. However, study has certain limitations. The all-male sample limits the generalizability of findings to female students or other populations. Furthermore, the short intervention period precludes conclusions about the long-term sustainability of these changes. Future studies should explore these effects in diverse populations and over extended periods. Building on these findings, future research could investigate the neural and biochemical correlates of *Trimurti Dhyana Yoga* through imaging and biomarker studies. Longitudinal studies assessing the sustained impact of such interventions on personality and stress resilience would also provide valuable insights. Additionally, integrating yoga-based practices into medical education curricula could be explored as a strategy to enhance student well-being.

## Conclusion:

A six-week *Trimurti Dhyana Yoga* intervention significantly enhances *Sattva* and reduces *Rajas* and *Tamas* among medical students, reflecting a positive shift in personality traits. These findings highlight the potential of yoga-based interventions in fostering emotional resilience and adaptive personality traits in high-stress populations. Future research should continue to explore the integration of such practices into educational and clinical settings to maximize their benefits.
